# Common neural correlates of vestibular stimulation and fear learning: an fMRI meta-analysis

**DOI:** 10.1007/s00415-023-11568-7

**Published:** 2023-02-01

**Authors:** Nicola Neumann, Miquel A. Fullana, Joaquim Radua, Thomas Brandt, Marianne Dieterich, Martin Lotze

**Affiliations:** 1 German Center for Vertigo and Balance Disorders (DSGZ), University Hospital, Ludwig-Maximilians-Universität München, Munich, Germany; 2 Functional Imaging Unit, Institute of Diagnostic Radiology and Neuroradiology, University Medicine Greifswald, Walther-Rathenau-Str. 46, 17475 Greifswald, Germany; 3 Adult Psychiatry and Psychology Department, Institute of Neurosciences, Hospital Clinic, Barcelona, Spain; 4 Institut d’Investigacions Biomèdiques August Pi i Sunyer (IDIBAPS), CIBERSAM, University of Barcelona, Barcelona, Spain; 5 Institute of Psychiatry, Psychology and Neuroscience, King’s College London, London, UK; 6 Department of Clinical Neuroscience, Karolinska Institute, Stockholm, Sweden; 7 Department of Neurology, University Hospital, Ludwig-Maximilians-Universität München, Munich, Germany; 8 SyNergy-Munich Cluster for Systems Neurology, Munich, Germany

**Keywords:** Anxiety, fear conditioning, Functional magnetic resonance imaging, Vestibular system, Voxel-based meta-analysis

## Abstract

**Background:**

A bidirectional functional link between vestibular and fear-related disorders has been previously suggested.

**Objective:**

To test a potential overlap of vestibular and fear systems with regard to their brain imaging representation maps.

**Methods:**

By use of voxel-based mapping permutation of subject images, we conducted a meta-analysis of earlier functional magnetic resonance imaging (fMRI) studies applying vestibular stimulation and fear conditioning in healthy volunteers.

**Results:**

Common clusters of concordance of vestibular stimulation and fear conditioning were found in the bilateral anterior insula cortex, ventrolateral prefrontal cortex and the right temporal pole, bilaterally in the adjacent ventrolateral prefrontal cortex, cingulate gyrus, secondary somatosensory cortex, superior temporal and intraparietal lobe, supplementary motor area and premotor cortex, as well as subcortical areas, such as the bilateral thalamus, mesencephalic brainstem including the collicular complex, pons, cerebellar vermis and bilateral cerebellar hemispheres. Peak areas of high concordance for activations during vestibular stimulation but deactivations during fear conditioning were centered on the posterior insula and S2.

**Conclusions:**

The structural overlap of both networks allows the following functional interpretations: first, the amygdala, superior colliculi, and antero-medial thalamus might represent a release of preprogramed sensorimotor patterns of approach or avoidance. Second, the activation (vestibular system) and deactivation (fear system) of the bilateral posterior insula is compatible with the view that downregulation of the fear network by acute vestibular disorders or unfamiliar vestibular stimulation makes unpleasant perceived body accelerations less distressing. This also fits the clinical observation that patients with bilateral vestibular loss suffer from less vertigo-related anxiety.

**Supplementary Information:**

The online version contains supplementary material available at 10.1007/s00415-023-11568-7.

## Introduction

There is increasing evidence, based on animal models and studies in humans, that the vestibular system is connected via multiple pathways with the functional structures relevant for fears and anxiety and cognitive and emotional effects [1–5]. These pathways between regions relevant for vestibular function and those relevant for fears and anxiety are particularly related to thalamocortical and cerebellar networks. The functional regulation is based on intrinsic neurotransmitters of the inner ear, thalamocortical and limbic connections. For instance, corticotropin-releasing factor mediates stress, fear and anxiety-related (loco)motor behaviors via the lateral vestibular nucleus [6]. This demonstrates the functional link between the anxiety and vestibular system since the vestibular nuclei are an integrative part of the balance system. Altogether there are close functional connections between stress, fear, anxiety, and balance, even at the level of the pontomedullary vestibular nuclei of the caudal brainstem.

Clinically, this is evident in patients with vestibular disorders showing a high comorbidity with anxiety, somatoform and depressive disorders such as in Menière’s disease (38–48%), vestibular migraine (49%) and vestibular paroxysmia (51%) [1, 7–9]. The extent of the fear and anxiety associated with vertigo and dizziness correlated with the extent of vestibular excitation or inhibition related to the vestibular disorder [2]. A recent survey on a total of 7083 patients with the key symptoms of vertigo, dizziness, and balance disorders confirmed that vestibular disorders characterized by an excitation or a tone imbalance of bilateral vestibular function were associated with increased anxiety related to vertigo and dizziness [10]. In contrast, patients with unilateral or bilateral vestibulopathy (BVP), i.e., with a loss of peripheral vestibular input, exhibited lower rates of associated anxiety about falling [10] or susceptibility to fear of heights despite postural instability [11]. The above described interrelations raised the question whether an intact vestibular function is required to develop vertigo-related anxiety [2, 10].

The vestibular and fear/anxiety networks can be artificially stimulated in healthy volunteers to allow their examination in an experimental brain imaging setting. For vestibular stimulation, caloric, galvanic or sound-induced otolith stimulation are used inside the scanner. The central vestibular network in humans identified by earlier brain imaging studies runs from the vestibular brainstem nuclei via the posterolateral and centromedial thalamus to parietotemporal cortex areas (meta-analyses: [12, 13]). Its multisensory core region representing the human homologue of the parieto-insular vestibular cortex in monkeys (PIVC) [14] is localized in the posterior insular gyri, the retroinsular area Ri, and the posterior parietal operculum [12, 13, 15]. Imaging of the anxiety network revealed a similarly extended distribution from brainstem to cortex with an overlap of the neuronal circuits of fear [16], which justified a commonly used model to investigate fear and anxiety by classical fear conditioning. Whereas anxiety is a non-stimulus directed general feeling, fear can be attributed to a certain aversive stimulus and is used in fear conditioning experiments. Thereby a stimulus (CS+), after being effectively paired with an aversive and therefore threatening stimulus, is compared against a comparable unpaired stimulus (CS−) (for methodological issues see [17]). In previous meta-analyses/reviews on fear conditioning fMRI studies, an extended fear network was described [18–20]. Using an image and coordinate-based meta-analytical approach (effect-size signed differential mapping), Fullana et al. [21] reported multiple clusters of activation, which can be assigned to the following functional subsystems relevant for the unique experience of fear and anxiety (see also [22, 23]): areas processing emotional arousal (thalamus, brainstem, hypothalamus) and attention (cingulate cortex, precuneus), areas associated with emotional anticipation and internal state regulation (anterior insula), emotional meaning and valence (ventrolateral prefrontal cortex, ventral striatum), and areas involved in motor reaction (premotor cortex, supplementary motor area) and conditioned learning (lateral cerebellar hemisphere).

A close interconnection between the neural circuits of vestibular and anxiety systems has been postulated before [3, 5], but not formally investigated yet. Possible anatomical links include networks from the brainstem (especially the parabrachial nucleus) [3, 24], cerebellum [4, 5], hypothalamus [3], thalamus [25], and amygdala up to the vestibular cortex [26] and other cortical areas subserving sensorimotor functions and emotional, cognitive, and visceral responses.

In the current study, we aimed to statistically overlap the neural substrates processing fear conditioning and vestibular stimulation, thereby confirming theoretical considerations derived from patient and animal studies. In a first step, we applied seed-based d-Mapping (SDM) on functional mapping studies in healthy volunteers investigating vestibular stimulation. The same SDM approach had been adopted for calculating meta-analytic data on fMRI studies applying fear conditioning that were provided by Fullana et al. [21]. Including actual statistical voxel-wise brain maps, SDM showed better results than previous coordinate-based meta-analyses with respect to overlap and sensitivity [27]. In a second step, we combined the two meta-analyses on functional imaging studies applying fear conditioning or vestibular stimulation and calculated peak areas of high convergence. In accordance with previous fMRI studies and meta-analyses (see above, [12, 13, 21]), convergence was expected in the anterior insula and secondary somatosensory cortex (S2), the prefrontal and parietal cortex, anterior cingulate cortex, secondary motor areas, amygdala, thalamus, basal ganglia, cerebellum and brainstem.

## Methods

### Literature search and study selection: vestibular stimulation

**Fig. 1:**
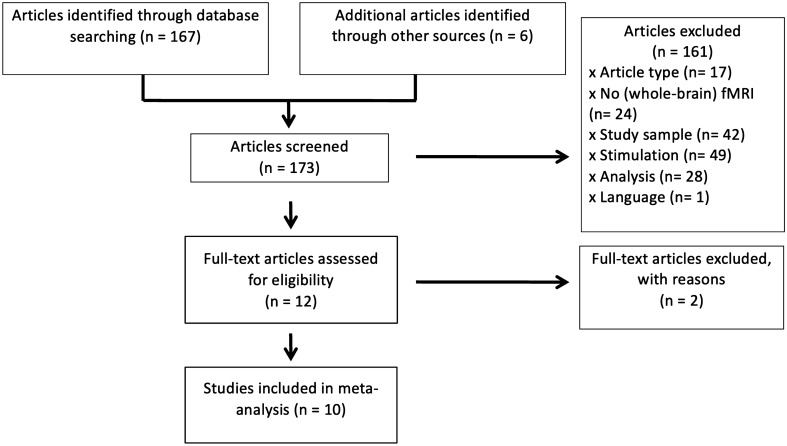
PRISMA diagram of study selection process. Studies were excluded, if they were reviews or meta-analyses (*n* = 17), if there was no brain imaging (*n* = 7), or brain imaging conducted with positron emission tomography (see above, *n* = 7), near infrared spectroscopy (*n* = 1), magnetic resonance spectroscopy (*n* = 1), diffusion tractography imaging (*n* = 6), if the study did not investigate the whole brain (*n* = 2), if the study investigated only patients/single cases (*n* = 41) or special groups (astronauts *n* = 1), if there was no galvanic or caloric stimulation (*n* = 37) or a concurrent task or visual stimulation (*n* = 12), if there was no random-effects analysis (*n* = 8), or those which investigated only resting state functional connectivity (*n* = 10), brain structure (*n* = 2), or magnetic stimulation inside the scanner (*n* = 8). One article was excluded because it was written in Chinese. If the study measured, but did not report results of the whole brain in common stereotactic space (*n* = 4), authors were contacted for further information. Of those, two were able to provide further data, the other two were discarded

A comprehensive literature search using PubMed was conducted for peer-reviewed fMRI studies on vestibular stimulation until September 3rd, 2021. The search terms were “vestibular cortex” OR “vestibular stimulation” OR “caloric stimulation” OR “galvanic stimulation” AND human AND fMRI”. Additionally, we checked the reference sections of published articles, particularly the two existing activation likelihood estimation (ALE) meta-analyses [12, 13] identifying another six publications. Altogether, 173 studies were identified. We focused on studies investigating healthy participants with galvanic or caloric vestibular stimulation, and conducted functional imaging of the whole brain as well as a 2nd level random-effects analysis. In the literature search, we did not include studies conducted with positron emission tomography, because of its generally lower spatial resolution. Sound-evoked vestibular stimulation studies (a combination of acoustic and otolith stimulation via the sacculus, see [28, 29] were excluded here for the following reasons: this kind of stimulation has to be a suprathreshold auditory one, which elicits an unnatural and incomplete stimulation of one type of vestibular receptors (sacculus). An evaluation as to vestibular excitation requires subtraction of the auditory effects. The remaining activation of the vestibular network is minimal and locally restricted according to the limited contribution of the sacculus input to a gross vestibular input. Furthermore, some of the more recent studies have methodological shortcomings, in particular a subthreshold auditory stimulation, which makes a concurrent vestibular stimulation questionable. Detailed reasons for exclusion of studies are provided in Fig. 1 and its legend.

Finally, we were able to collect imaging results from 10 independent fMRI studies using vestibular stimulation with 187 healthy adult participants (75 males, 74 females, and 38 of unclear sex, mean age 32.7 years) (Fig. 1, Table 1). All but one study reported the contrast vestibular stimulation versus baseline/rest condition. All corresponding authors were contacted and asked for the original group-level statistical t-maps, which were obtained for eight studies. For the remaining two studies, peak coordinates were extracted and coded from the original publication or from supplementary data provided by corresponding authors. Eight of ten selected studies were based on galvanic vestibular stimulation, which induced a gentle perception of body sway without distressing adverse effects. The caloric vestibular stimulation as used for clinical routine is only mildly aversive in some individuals. Subjects with unpleasant side effects were, as a rule, not included in experimental studies.

**Table 1:** Characteristics of 10 fMRI studies with stimulation of the vestibular system included in the meta-analysis

References	*N*	% males	Mean age (SD)	Type of stimulation	Ear	Stimulation duration	Independent assessment of stimulation	Contrast	Software package	Stereotactic space
Becker-Bense et al. [15]	19	42	47 (10)	GVS AC 1 Hz, 1.75–2.5 mA	Bipolar	4 blocks à 30 s	Subj. vestibular sensations	GVS vs. rest	SPM 12	MNI
Della-Justina et al. [34]	21	57.1	26.9 (3.9)	GVS AC 1 Hz, av. 1.5 mA	Bipolar	4 blocks à 21 s	Subj. vestibular sensations	GVS vs. rest	SPM 8	MNI
Helmchen et al. [35]	27	52	62.3 (11.8)	GVS AC 1 Hz, 1.5 mA above perception threshold	Bipolar	12 blocks à 12 s	Perceived motion intensity	High GVS vs. sham	SPM 12	MNI
Indovina et al. [40]	17	41.2	27 (6)	CAL, 10 °C	Both ears successively	3 tests per ear, 60 s	Subj. vestibular sensations	CAL vs. baseline	SPM 2	MNI
Russo et al. [41]	12	42	28.7 (6.7)	CAL, 4 °C	Left	1 test, 1.5 s	Subj. vestibular sensations	CAL vs. control	Brain Voyager QX	TAL
Stephan et al. [36]	21	47.6	29.1 (5.7)	GVS AC 0.1–5 Hz, ± 2.5 mA	Bipolar	3 blocks à 22.5 s	Perceived motion	GVS vs. rest	SPM 99	MNI
Stephan et al. [37]	28	Not stated	25	GVS DC (model 2, transient)	Bilateral	20 blocks à 21 s	Subj. vestibular sensations	GVS vs. rest	SPM 5	MNI
Suzuki et al. [42]	10	Unclear	Range 25–38	CAL, 4 °C	Both ears successively	2 blocks à 30 s	Subj. vestibular sensations	CAL vs. baseline	SPM 99	TAL
zu Eulenburg et al. [38]	16	50	26.3 (3)	GVS AC 1 Hz, 3 mA	bipolar	6 blocks à 25 s	Unpleasantness rating	GVS vs. rest	SPM 5	MNI
zu Eulenburg et al. [39]	16	50	27.1	CAL, 20 °C	Both ears successively	5 tests per ear, 24 s	Intensity and unpleasantness rating	CAL vs. rest	SPM 5	MNI

All participants were healthy

*GVS* galvanic vestibular stimulation, *CAL* caloric stimulation, *AC* alternating current, *DC* direct current, *SPM* statistical parametric mapping, *MNI* Montreal Neurological Institute, *TAL* Talairach

### Literature search and study selection—fear conditioning

Literature search and study selection were carried out as described in detail by Fullana et al. [21]. After excluding ineligible studies, 27 studies with 677 participants (366 males, mean age 25.4 years) using a delay differential cue-conditioning paradigm were obtained. All studies reported a CS +  > CS−, 19 of them also the opposite contrast. For 13 of them, original empirical 3D statistical images were available [21]. The entire data of fear conditioning have been published before in [21].

### Meta-analytic procedure

Functional activation differences between the stimulation of the vestibular system and the control (rest) condition were analyzed using “Seed-based d-Mapping-Permutation of Subject Images (Sdm-Psi)”, version 6.21 (https://www.sdmproject.com) [30]. In contrast to previous coordinate-based meta-analyses testing convergence of the reported peaks [31], this method assigned effect sizes to each voxel. When original 3D statistical images were available, it converted t-values to effect sizes [32]. If only peak information was available, effect sizes were imputed multiple times based on the known data (e.g., effect size of the peaks) and the spatial covariance between adjacent voxels [33]. These study images were then voxel-wise combined into random-effects meta-analysis images. To assess the statistical significance using FWE correction, SDM-PSI imputed and permuted subject images saving maximum threshold-free cluster enhancement (TFCE) statistic of the combined meta-analysis image from each permutation [30].

We used the Sdm-Psi pipeline with default parameter settings. After preprocessing, the calculated peak of one original dataset differed from the reported peak in the respective paper, so that instead of the statistical image, the reported peak information was included to the analysis. Our analysis thus comprised seven original statistical images [15, 34–39] and three studies with peak information [40–42]. Meta-analytic means were calculated using 50 imputations, followed by FWE correction with 1000 permutation. TFCE-corrected maps were thresholded with *p* < 0.05. MNI coordinates of the outcome files were checked and specified with the “Anatomy” toolbox (vs. 2.2b, [43]) of Statistical Parametric Mapping (SPM 12). The *I*^2^ index and Egger’s method were used to assess for heterogeneity of effect sizes and publication bias (robustness analyses, see Supplementary Table 1).

### Conjunction analysis

To assess the brain regions of common activations of vestibular stimulation and fear conditioning, we conservatively selected the voxels that showed statistical significance in both the vestibular and the fear conditioning meta-analysis, while we discarded voxels that showed statistical significance in only one of the meta-analyses. The statistical significance was not derived from the *z* value, but rather from the meta-analyses’ statistical significance (e.g., derived from the TFCE-based permutation test).

To create the map of *z* values, we conservatively assigned each voxel the lowest *z* value between both meta-analyses (i.e., that closer to zero). For instance, if in a given voxel, the *z* value was 3.2 in the vestibular meta-analysis and 3.7 in the fear conditioning meta-analysis, we would use 3.2 for the conjunction analysis. We thus provide a map of the minimum effects across meta-analyses: for each voxel, the *z* value in the different meta-analyses is at least as high as the *z* value of this map.

Visualization of activation maps was performed using MRICroGL (64bit OSX; Version 2016).

## Results

### Neural correlates of vestibular stimulation

**Fig. 2:**
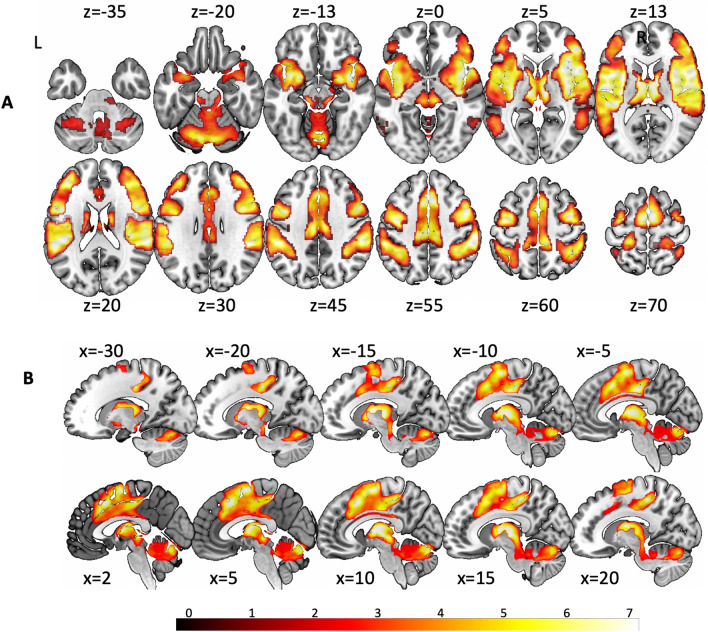
Peak areas of high concordance during vestibular activation overlaid on T1-MNI reference brains in yellow–red (color coded *z* values on the bottom). Axial/transversal slice position is plotted as *z*-coordinates for the MNI-system. **A** Axial slices with representations comprising the bilateral insula and adjacent structures (right temporal pole, ventrolateral prefrontal cortex/VLPFC, putamen), thalamus, right amygdala, right Heschl’s gyrus (primary auditory cortex/A1), frontal lobe (dorsolateral PFC, left orbitofrontal cortex, cingulate cortex/CC, supplementary motor area/SMA, premotor cortex/PMC, primary motor cortex/M1), parietal areas (primary and secondary somatosensory cortex/S1 and S2, supramarginal gyrus, inferior and superior parietal lobe/IPL and SPL), cerebellar dentate nuclei, hemispheres and vermis, and ponto-mesencephalic brainstem with superior and inferior colliculi. **B** Cluster of high concordance around the brainstem showed activation in thalamus, PAG, nucleus ruber, dorsal midbrain including superior colliculi, vermis, and posterior cerebellar hemispheres. Slices have been positioned in x = −30; −20, −15, −10, −5, 2, 5, 10, 15, 20

The brain regions demonstrating significant peak areas of high concordance during activation of the vestibular network showed the bilateral insula and adjacent structures, right Heschl’s gyrus (A1), the right temporal pole, putamen, thalamus, right amygdala, dorsolateral prefrontal cortex (DLPFC), left orbitofrontal cortex (OFC), cingulate cortex (CC), supplementary motor area (SMA), premotor cortex (PMC), M1, S1, S2, supramarginal gyrus, inferior parietal lobule (IPL), superior parietal lobule (SPL), cerebellum (vermis, dentate nuclei, hemispheres), and ponto-mesencephalic brainstem with inferior and superior colliculi (Fig. 2A, Supplementary Table 1) (for medial structures and brainstem see Fig. 2B).

Peak areas of high concordance of deactivation under vestibular stimulation was evident in a cluster (1375 voxels) in the bilateral middle orbital gyrus (MNI: 4, 42, − 4; SDM-Z = 4.55, *p* = 0.001; MNI: − 6, 50, − 4; SDM-Z = − 3.5, *p* = 0.004) and left anterior cingulate cortex (MNI: − 6, 40, 2; SDM-Z = − 3.93, *p* = 0.004; see Supplementary Fig. 1). A complete table showing all significant coordinates is added to the Supplemental Material (Supplementary Table 1).

Our additional analyses indicated that there was low heterogeneity (mean *I*^2^ = 18.1%, sd = 18.7, range 0.02–57%) and no evidence of potential publication bias (Egger’s test: 6 out of 44 significant with *p* < 0.05) in the main results (Supplementary Table 1).

### Regions of high concordance across vestibular stimulation and fear conditioning

**Fig. 3:**
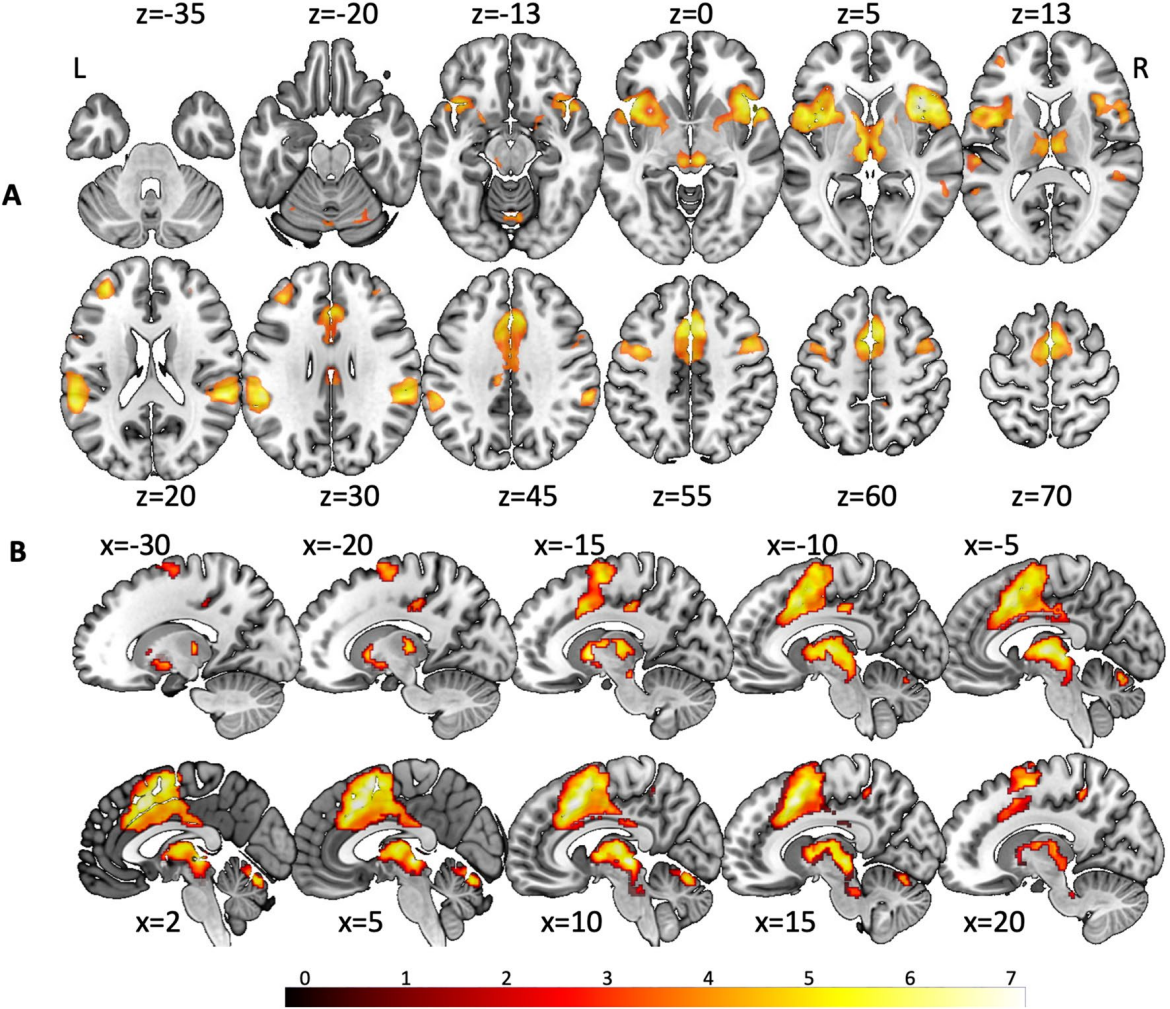
Brain regions showing significant high concordance for vestibular stimulation (analysis presented here) and fear conditioning (meta-analysis published in [21]). Color bar provides *z* value coding. Z-position plotted above each axial/transversal slice. **A** For axial slice overlay of the conjunction analysis between the two conditions activations were significant for the anterior insula, frontal lobe areas (dorso- and ventrolateral PFC, dorsal anterior cingulate cortex/medial cingulate cortex, the supplementary motor area/SMA and the premotor cortex/PMC), subcortical structures (antero-medial thalamus, anterior thalamic projections, striate, mesencephalic brainstem, cerebellar vermis and parts of the posterior hemispheres), temporal areas (Heschl gyrus; superior and middle temporal gyrus), and parietal areas (S2, IPL). **B** The cluster of concordance around the brainstem showed activation in the thalamus, periaqueductal gray, nucleus ruber, dorsal midbrain, vermis, and posterior cerebellar hemispheres. Overall, all areas of high concordance detected for vestibular activation in the brainstem, thalamus and cerebellum were also active during fear conditioning

Regions that showed high concordance during vestibular stimulation and fear conditioning included the bilateral anterior insula in a common cluster with the VLPFC and the temporal pole, the DLPFC, cingulate cortex, supplementary motor area, premotor cortex, Heschl’s gyrus, superior and middle temporal gyrus, S2, IPL, and subcortical structures such as the antero-medial thalamus, anterior thalamic projections, bilateral putamen, mesencephalic brainstem, cerebellar vermis and posterior hemispheres (Fig. 3). Supplementary Table 2 provides a more detailed description of peak coordinates and areas.

**Fig. 4:**
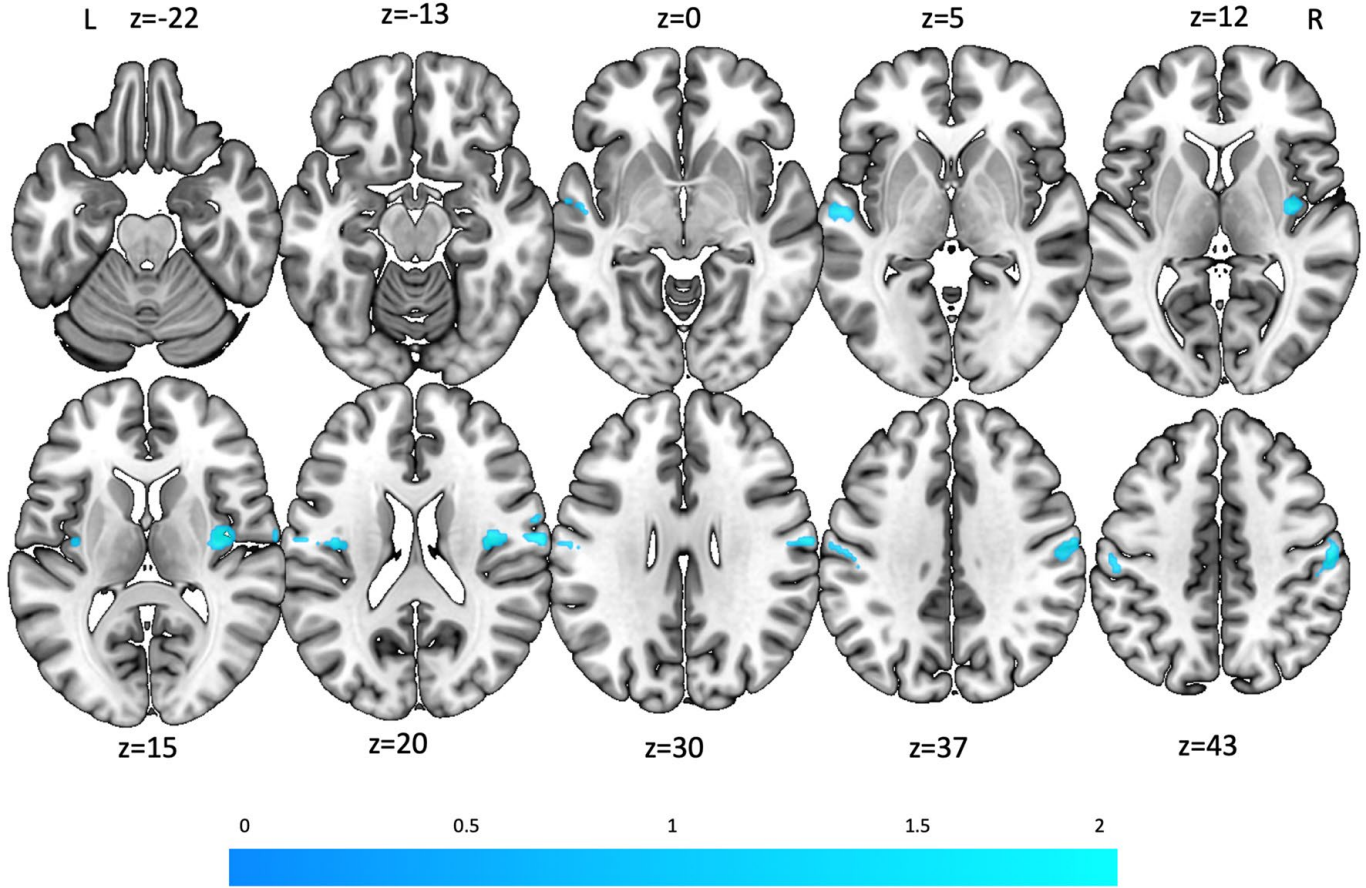
Regions of concordance for activation during vestibular stimulation and deactivation during fear conditioning: right postcentral gyrus, bilateral insula (predominantly anterior long insular gyrus, i.e., first long insular gyrus according to [52], secondary somatosensory cortex/S2 (predominantly OP1 and OP3), and left superior temporal gyrus (color bar provides *z* value coding). Axial/transversal slice position is plotted as *z*-coordinates for the MNI-system

The calculation of areas of concordance for activation during vestibular stimulation and deactivation for fear conditioning revealed significant results in the right postcentral gyrus (MNI: 66, − 10, 22, SDM-Z = 3.23, 322 voxels), the bilateral posterior insula (anterior part of the long insular gyrus; MNI: 38, − 10, 16, SDM-Z = 2.58, 259 voxels; MNI − 34, − 10, 16, SDM-Z = 2.34, 235 voxels), the bilateral S2 (OP1 and OP3), and the left superior temporal gyrus (MNI: − 56, − 12, − 2, SDM-Z = 1.93, 73 voxels) (Fig. 4). Note that the statistical significance for the conjunction analysis was not derived from the *z* values, but rather from the two meta-analyses’ statistical significance.

The reverse contrast of activation for fear conditioning and deactivation during vestibular stimulation did not yield any significant result. The calculation of areas of concordance for deactivation under vestibular stimulation and deactivation for fear conditioning showed a small region in the left superior frontal gyrus (MNI: 0, 54, 12; SDM-Z = 1.92, 45 voxels).

## Discussion

Applying a voxel-based meta-analysis across 10 functional imaging studies during vestibular stimulation, we identified a distributed network comprising the bilateral insula and adjacent structures, Heschl’s gyrus and superior temporal lobe, anterior and medial thalamus, right superior amygdala, frontal lobe structures (VLPFC, DLPFC, AVCC, MCC, SMA, PMC), superior and inferior parietal lobe and the intraparietal sulcus, mesencephalic brainstem and cerebellum (vermis and anterior hemisphere). This is in agreement with several earlier human imaging studies conducted by various groups [12, 13, 25]. With regard to our main question about a functional overlap of brain areas, which show an overlap during processing of vestibular stimulation and fear conditioning, we found convergence in the lateral and medial prefrontal cortex, secondary motor areas, the anterior-medial insula, superior temporal lobe including auditory processing areas, and subcortical areas such as the thalamus, the basal ganglia, the mesencephalic brainstem, cerebellar vermis and posterior cerebellar hemispheres. Further, we identified opposite effects of both kinds of stimulation: peak areas of high concordance for activations during vestibular stimulation but deactivations during fear conditioning were centered on the posterior insula and S2.

In the following the most relevant areas of a network overlap will be discussed with respect to their possible functional implications. Three main findings regard the insula: (A) the entire insula in both hemispheres was mapped demonstrating significant activation during vestibular stimulation, (B) the conjunction of vestibular and fear conditioning stimulation revealed an overlap of activation in the anterior insula bilaterally, (C) the posterior insula showed concordance of increased activation during vestibular stimulation but decreased activation during fear conditioning. Overall, the insula is called the hub of the salience network integrating incoming stimuli of all modalities with the internal state of the body and mind (i.e., interoception) [44, 45]. The multisensory posterior insula—highly interconnected with the thalamus, other vestibular as well as primary and secondary somatosensory cortex [46]—is involved in the discrimination of body motion and provides topographic and modality-specific interoceptive signals to the anterior insular cortex. It also comprises the core region of the vestibular cortical network (e.g., PET imaging: [47]; structural connectivity: [48]; stroke lesion and balance impairments: [49]). In a posterior to anterior progression, an integration of these visceral and somatosensory information takes place going along with interoceptive awareness and subjectivity [44, 45]. The anterior ventral insula represents a socioemotional region [50] connected to the orbitofrontal cortex as well as the ACC [46] with descending influences on the amygdala and autonomic nervous system, which mediate fear and anxiety systems [16, 51]. Notably, especially the discriminative posterior insula activated during vestibular processing is downregulated during fear conditioning, which may make unfamiliar distressing body accelerations or acute vestibular disorders less threatening. This underlines and extends hypotheses raised before on the basis of patient investigations with a decrease of vertigo-related anxiety in bilateral or unilateral peripheral vestibular loss [2, 10]. This interpretation is based on the application of a fundamental concept discovered as a reciprocal inhibitory interaction between sensory systems: experimental activations of the vestibular cortical network were associated with concurrent deactivations of the visual and somatosensory cortex areas [52–54, for review: 55]. Originally this pattern of reciprocal activations and deactivations were found during visually induced self-motion perception, for example, activations of occipital and parietal visual areas were associated with deactivations of the multisensory vestibular cortex [56, 57]. The functional interpretation was that during motion perception, the dominant sensorial weight may be shifted from one modality to the other, thereby resolving conflicts of a sensory mismatch. Later, such interactions were found for other sensory modalities, e.g., the somatosensory and nociceptive, the nociceptive and the vestibular, the tactile sensory and visual, and the visual and auditory systems [52, 58–60]. Although in the current study, we did not effectively investigate an interaction between the vestibular and fear systems, the activation–deactivation of the posterior insula based on two meta-analyses might support the interpretation presented above. It might further extend another functional implication described before on the basis of patient investigations that showed a decrease of vertigo-related anxiety in bilateral or unilateral peripheral vestibular loss [2, 10].

Convergent activation in S2 (OP1) indicated an important role in both hemispheres not only for vestibular processing [12, 13, 15], but also for fear conditioning [21]. When contrasting activation during vestibular stimulation with deactivation during fear conditioning, predominately OP1 and OP3 were activated. These areas are adjacent to the dorsal posterior insula and close to area Ri, which showed high fMRI response to vestibular stimulation in an earlier meta-analysis by [12]. This confirms studies in which several neighboring areas in the insular-opercular cortex were reported to process cortical vestibular input such as Ri, Ig, OP1, 2, 3, and 4 [12, 61, 62].

The role of the amygdala during fear conditioning has been the subject of much discussion. Since amygdala activation is stronger during the first part of fear conditioning experiments [63], and also only present for a very short time following an aversively rated stimulus [23], it had often not been identified in fear conditioning studies [20, 21, 64, 65]. Nowadays, the role of the amygdala is discussed as a “significance indicator” rather than a specific indicator for fearful stimuli [22]. Our current study identified convergence of amygdala activation during vestibular processing, which might well fit into the notion that vestibular input is involved in avoidance behavior (see also Discussion on cingulate cortex and collicular complex).

High convergence was also evident in the dorsal anterior cingulate cortex for both vestibular and fear conditioning, compatible with the major involvement of this region in emotion by connections of the anterior part to the orbitofrontal cortex and the amygdala, its tight interconnection with the anterior insula [46], but also interactions with higher cortical areas processing spatial orientation (posterior part of the parietal cortex [66]) and memory (hippocampal system). The motor output of the midcingulate cortex is interconnecting secondary motor areas (for review see: [67, 68]). After the amygdala indicated high relevance of a stimulus, the cingulate cortex is associated with increasing attention and, for its superior more dorsal areas, the initiation of behavioral consequences (retreat). Therefore, the dorsal anterior cingulate cortex has been described to be essential in processing the “tipping point” between approach and avoidance behavior, relating to the probability of an aversive outcome [69]. Resulting motor behavior is then organized in a more profound motor program processed in the SMA and the dPMC. Both types of stimuli were relevantly activating the medial cingulate cortex, the SMA and the dPMC as secondary motor areas. The dorsolateral prefrontal lobe contributes to increased higher cognitive functioning when planning processes following aversive stimulation but also the cognitive control of emotional retreat in an experimental condition (for a discussion see also [64]).

With regard to the overlap between vestibular and fear processing, we also found convergence in the antero-medial thalamus, anterior thalamic projections, and the striatum (especially left ventral putamen). These thalamic areas are interconnected with areas of the prefrontal and the temporal lobe, which showed also significant convergent activation. Both fear and vestibular stimulation provide relevant behavioral input resulting in an increased arousal, which is processed in the thalamus [70]. In short, with respect to function one could assume that the posterolateral thalamic vestibular projections constitute the primary input, whereas the anterior thalamic projections are involved in more complex behavioral processing including emotions and anxiety.

Convergence for vestibular stimulation and fear conditioning was detected in the cerebellar vermis and posterior cerebellar hemisphere (Larsell’s VI), but not in sensorimotor areas of the anterior cerebellar hemisphere (Larsell HV). Posterior superior cerebellar activation has been shown to be involved in fear conditioning [64], and also occurred during vestibular stimulation in the current meta-analysis.

The current meta-analysis of vestibular stimulation studies exhibited convergent bilateral activation of the inferior colliculi (IC) and the superior colliculi, functionally most relevant structures for eye-, head- and body orientation and motor behavior in space and largely dependent on life-threatening emotional processes. The IC is a center for integration and analysis of various auditory features with ascending and descending pathways in the brainstem transmitting auditory information to the ipsilateral thalamus and cerebral cortex [71]. For 3D-sound localisation, the vestibular system may provide the three-dimensional spatial frame of the current head position in space. Animal studies have shown vestibular input to the IC [72–75]. Thus, the IC is a multisensory hub with major auditory, but also vestibular input as found in the current study.

The superior colliculus is a multisensory nucleus with the major input from the retina as well as auditory and somatosensory receptors to mediate the orientation of the head and senses toward targets of interest to analyze (sensory input) and control (motor output) gaze as well as ears, head, and body in space. This synkinesis of coordinated orientation movements is critical for survival. In a threatening situation, the superior colliculus elicits a reflex-like orientation and defense response in animals (for review see: [76–78]). After recognition of a threatening event the decision to either fight or fly is likely to depend on a thalamo-cortical arousal, which releases an appropriate preprogramed sensorimotor pattern. In humans, it is similarly critical, e.g., for avoidance of imminent collisions during driving or in sports, shown by Billington and co-workers [79] in an fMRI investigation in which the SC and the pulvinar nucleus of the thalamus were activated. Although not yet described, interconnection with vestibular input is imperative and has been shown in the current meta-analysis on vestibular stimulation (Fig. 2). Accordingly, the current overlap with fear conditioning (Fig. 3) revealed that the SC is also part of the anxiety network. In particular, this activation documents the possible link between vestibular and anxiety networks.

Beyond this, anxiety and vegetative dysregulation are not only symptoms of acute vestibular vertigo syndromes but can also be the major provoking stimuli of frequent dizziness/vertigo syndromes like functional phobic postural dizziness (or PPPD) [80, 81] or visual height intolerance/acrophobia [82]. That irrational anxiety of falling rather than the perceived visual height is the causative factor of postural imbalance and height intolerance has also been shown by others [83, 84].

### Limitations

There are a number of limitations of the current study: first, we calculated the overlap of two meta-analyses that had different sample sizes and thus different power, so that the meta-analysis with the smaller sample (vestibular stimulation) may have detected fewer regions of convergence than fear conditioning and accordingly reduced the overlap. It is worth reminding here that the two meta-analyses were independent, i.e., they were conducted with different participants at different time points. Second, every meta-analysis can just be as exact as the studies selected as their basis. This restricts conclusions on brainstem activation (not optimized methodological procedures for brainstem imaging or high spatial resolution imaging), the modeling of amygdala response and other functional activations sensitive to timing, but also the application of cluster-based statistical thresholding. An exact localization of processes in the brainstem has therefore to be performed in fMRI studies optimized for high brainstem resolution handling specific problems, such as liquor pulsation, high susceptibility artifacts and spatial normalization problems when an MNI brain normalization is applied (for an early investigation of brainstem functional activation in general see [85]; for an attempt optimizing brainstem fMRI see [86]).

In addition, whereas fear conditioning is perfectly balanced for the parameter fear induction, since CS+ and CS− differ only with regard to their association with an aversive stimulus in the past, vestibular stimulation paradigms usually contrast the stimulation condition versus baseline (but see also [35, 52]). Therefore, activation maps in vestibular stimulation studies are much more extended and activation intensity increased compared to a specific CS+ /CS− contrast. Thus, a direct contrast between meta-analytic representation maps for each condition was not conducted in the current study. Related to this, non-specific effects of vestibular stimulation may have contributed to the activation patterns in the original fMRI studies.

## Conclusions

Earlier electrophysiological and tracer studies in animals and fMRI/ PET imaging in humans have established the concept of complex bilateral cerebral networks for the vestibular (for review [48, 72, 87]) and the fear/anxiety systems (for review: [3, 21]). Both networks extend from the caudal pontine brainstem, the vestibular nuclei for the vestibular system [48, 72] and the parabrachial nuclei for the fear/anxiety system [88] to multiple cortex areas. The networks have distinct structures; their operations may be separate or interactive. The current meta-analysis of the neural correlates of vestibular excitation and fear conditioning and their overlap disclosed the brain regions associated with both functions. This is in line with the clinical finding of psychiatric comorbidity and vertigo-related anxiety being maximal with vestibular excitation and minimal with loss of vestibular function [2, 10]. Future studies should investigate the interaction between fear and vestibular circuits by manipulating both factors in a group of same individuals in a functional imaging study.

Regions exhibiting high overlap in vestibular stimulation and fear conditioning include the anterior insula and anterior cingulate sulcus, whose above discussed structural and functional features are essential for emotional interoception. The posterior cingulate sulcus and superior colliculus mediate multisensory spatial orientation and memory. The antero-medial thalamus with its connection to the prefrontal and temporal lobes and midbrain colliculi is involved in an increased arousal. This is relevant for avoidance of obstacles or other potentially dangerous situations during locomotion in unfamiliar terrains. The superior colliculus is the major hub for reflexive coordinated eye, head, body orientation movements, for instance, recognition and avoidance of unexpected threatening stimuli (fight or fly). The posterior insula and S2 are special in that the contrast for vestibular activations and fear conditioning deactivations resulted in a deactivation of these areas, possibly involved in weakening distressing anxiety and vegetative effects of excessive vestibular stimulations or acute vertigo disorders.

## Supplementary Information

Below is the link to the electronic supplementary material.

Supplementary file1 (TIF 2140 kb)

Supplementary file2 (PDF 95 kb)

Supplementary file3 (PDF 69 kb)

## Data Availability

Statistical maps of the peak areas of high concordance during activation and deactivation of the vestibular network and the conjunction of the meta-analyses of vestibular stimulation and fear conditioning are available under https://github.com/martinlotze/Metaanalysis_vestibular_fear
