# Supplementary figures and images for: Common neural correlates of vestibular stimulation and fear learning: an fMRI meta-analysis
Source: J Neurol. 2023 Feb 1;270(4):1843–56. doi: 10.1007/s00415-023-11568-7 (PMC10025232; doi:10.1007/s00415-023-11568-7)

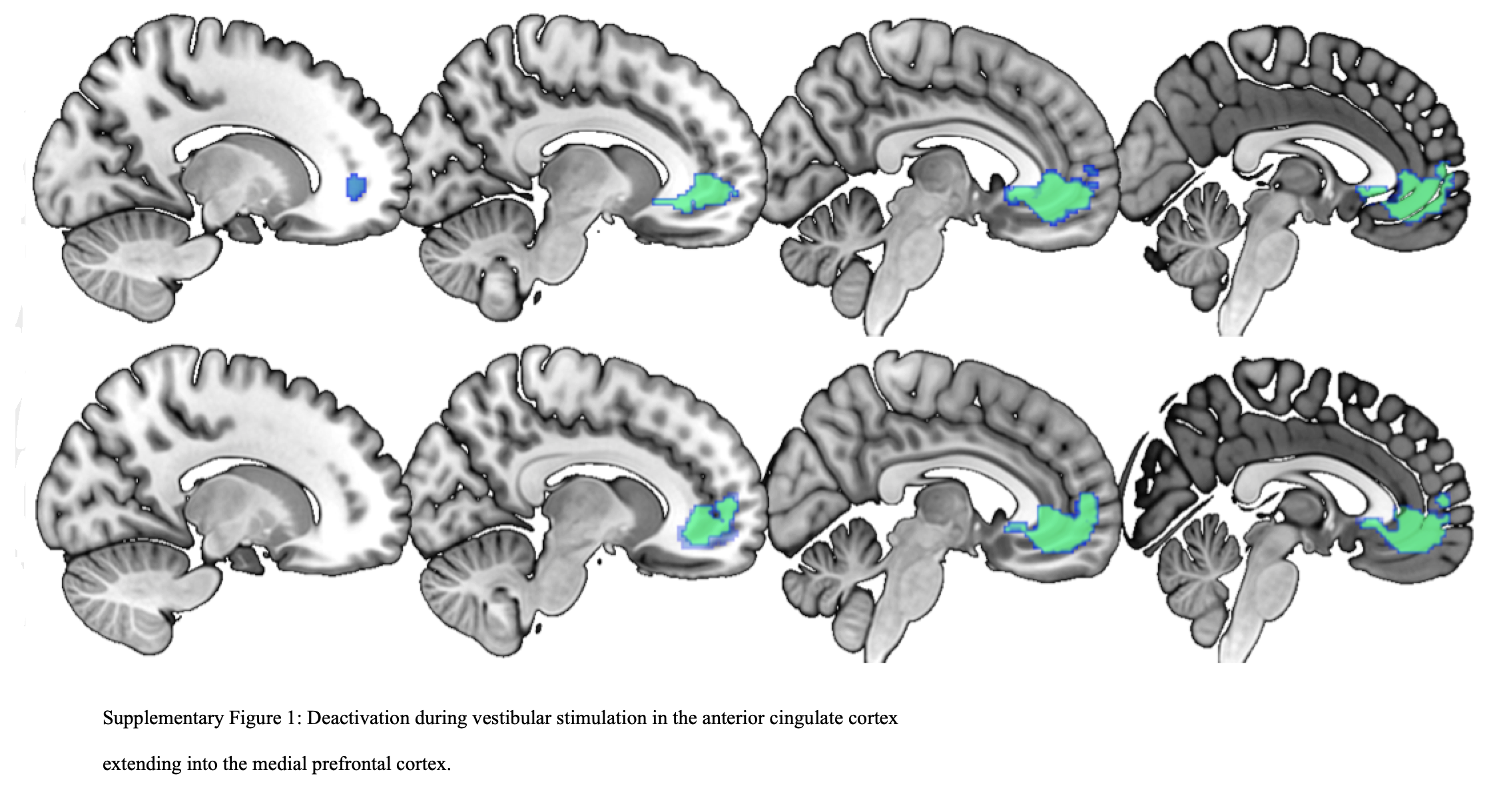

Supplement: Supplementary file 1 — Supplementary file1 (TIF 2140 kb) [file 415_2023_11568_MOESM1_ESM.tif]
