# Supplementary material for: Common neural correlates of vestibular stimulation and fear learning: an fMRI meta-analysis
Source: J Neurol. 2023 Feb 1;270(4):1843–56. doi: 10.1007/s00415-023-11568-7 (PMC10025232; doi:10.1007/s00415-023-11568-7)
Supplement: Supplementary file 2 — Supplementary file2 (PDF 95 kb) [file 415_2023_11568_MOESM2_ESM.pdf]

Supplementary Table 1: Results of the meta-analysis of the vestibular stimulation studies at p< 0.05.

| **Description** | | **Local peaks and breakdown** | | | **Hetero-geneity** | **Meta-bias** | | |
|---|---|---|---|---|---|---|---|---|
| | Hemisphere | MNI (x, y, z) | SDM-Z | p (TFCE corr) | $I^2$ | Egger's test | Brain region | No. of voxels |
| Insula | left | -32,2,12 | 8.467 | 0.001 | 2.07 % | 1.83, p= 0.39 | Anterior insula | 1500 |
| | | -30,8,8 | 7.545 | 0.001 | | | Anterior insula | |
| | | -42,4,2 | 6.319 | 0.001 | | | Anterior insula | |
| | | -34,-18,12 | 7.776 | 0.001 | 19.91 % | 2.49, p= 0.29 | Posterior insula, area lg2 | |
| | | -36,6,-12 | 7.700 | 0.001 | | | Posterior insula | |
| | | -40,-8,6 | 6.935 | 0.001 | | | Posterior insula | |
| | | -36,-2,-8 | 6.470 | 0.001 | | | Posterior insula | |
| | right | 36,10,8 | 8.858 | 0.001 | 2.84 % | 1.23, p= 0.61 | Anterior insula | 1550 |
| | | 46,10,-2 | 7.800 | 0.001 | | | Anterior insula, area 44 | |
| | | 38,22,2 | 7.513 | 0.001 | | | Anterior insula | |
| | | 44,20,-8 | 6.698 | 0.001 | | | Anterior insula | |
| | | 42,6,-8 | 8.701 | 0.001 | | | Anterior insula | |
| | | 34,12,4 | 8.701 | 0.001 | | | Anterior insula | |
| | | 40,2,-14 | 9.338 | 0.000000001 | 27.0 % | 3.17, p= 0.13 | Posterior insula | |
| | | 40,-18,2 | 7.338 | 0.001 | | | Posterior insula, area lg2 | |
| | | 40,-16,-2 | 7.220 | 0.001 | | | Posterior insula, area lg2 | |

| | | | | | | | | |
|---|---|---|---|---|---|---|---|---|
| Heschl's gyrus (A1) | right | 60, -4, 6 | 7.539 | 0.001 | 43.06 % | 3.93, p= 0.01 | Heschl's gyrus | 180 |
| Temporal | left | -48, -32, 16 | 8.326 | 0.001 | 38.3% | 3.79, p= 0.04 | Superior temporal gyrus | 1493 |
| | | -56, -38, 18 | 8.422 | 0.001 | | | Superior temporal gyrus | |
| | | -40, -16, -6 | 6.321 | 0.001 | | | Superior temporal gyrus | |
| | | -42, -12, -10 | 6.210 | 0.001 | | | Superior temporal gyrus | |
| | | -52, -54, 10 | 5.014 | 0.001 | 9.72% | -0.72, p= 0.81 | Middle temporal gyrus | 848 |
| | | -64, -50, -2 | 2.725 | 0.023 | | | Middle temporal gyrus | |
| | | -52, -60, 6 | 4.701 | 0.001 | | | Middle temporal gyrus | |
| | | -50, -64, 6 | 4.272 | 0.001 | | | Middle temporal gyrus | |
| | | -60, -60, -2 | 2.568 | 0.007 | | | Middle temporal gyrus | |
| | | -44, -52, -26 | 2.532 | 0.023 | | | Inferior temporal gyrus, area FG4 | |
| | | -60, -60, -6 | 2.227 | 0.038 | | | Inferior temporal gyrus | |
| | right | 62, -34, 18 | 7.946 | 0.001 | 39.09 % | 3.78, p= 0.08 | Superior temporal gyrus, area PFcm | 1223 |
| | | 48, -32, 14 | 5.944 | 0.001 | | | Superior temporal gyrus | |
| | | 50, -24, 16 | 6.518 | 0.001 | | | Superior temporal gyrus, area OP1 | |
| | | 24, 6, -22 | 3.328 | 0.003 | 57.20 % | 2.59, p= 0.39 | Temporal pole, superior temporal gyrus | 317 |
| | | 42, 20, -20 | 3.301 | 0.003 | | | Temporal pole, superior temporal gyrus | |
| | | 64, -52, -4 | 2.416 | 0.023 | | | Middle temporal gyrus | 438 |
| | | 56, -40, 8 | 3.891 | 0.001 | | | Middle temporal gyrus | |
| Inferior parietal lobule/ intraparietal sulcus | left | -56,-36,44 | 7.249 | 0.001 | 3.6 % | 1.74, p= 0.42 | Inferior parietal gyrus, area PFt | 1433 |
| | | -52, -36, 40 | 7.222 | 0.001 | | | Inferior parietal gyrus, area PFt | |
| | | -36, -48, 42 | 6.293 | 0.001 | | | Inferior parietal gyrus, area hlP1 | |
| | | -38, -48, 46 | 6.110 | 0.001 | | | Inferior parietal gyrus, area hlP3 | |
| | | -50, -42, 52 | 5.898 | 0.001 | | | Inferior parietal gyrus, area hlP2 | |
| | | -46, -42, 44 | 5.875 | 0.001 | | | Inferior parietal gyrus, Area hlP2 | |
| | | -44, -44, 48 | 5.866 | 0.001 | | | Inferior parietal gyrus, BA 2 | |
| | | -60, -44, 38 | 5.060 | 0.001 | | | Inferior parietal gyrus, area PFm | |

| | | | | | | | | |
|---|---|---|---|---|---|---|---|---|
| | | -48, -36, 24 | 7.322 | 0.001 | 53.0 % | 4.83, p= 0.04 | Supramarginal gyrus, area PFcm | 855 |
| | | -60, -22, 16 | 5.551 | 0.001 | | | Supramarginal gyrus, area OP1 | |
| | right | 40, -48, 50 | 6.274 | 0.001 | 0.02 % | 0.15, p= 0.95 | Inferior parietal lobule, area hlP3 | 858 |
| | | 58, -28, 42 | 8.485 | 0.001 | 10.4 % | 3.19, p= 0.04 | Supramarginal gyrus, area PFt | 1230 |
| | | 56, -32, 46 | 8.028 | 0.001 | | | Supramarginal gyrus, area PFt | |
| Cingulate cortex | left | -2,16,24 | 3.748 | 0.001 | 2.07 % | 1.83, p= 0.39 | Anterior cingulate | 288 |
| | | -4,8,28 | 3.748 | 0.001 | | | Anterior cingulate, BA 30 | |
| | | 0,20,24 | 3.748 | 0.001 | | | Anterior cingulate | |
| | | -6,-22,40 | 7.480 | 0.001 | 0.80 % | -3.4, p= 0.37 | Median cingulate | 1143 |
| | | -6,-24,44 | 7.277 | 0.001 | | | Median cingulate | |
| | | -10,-26,46 | 7.000 | 0.001 | | | Median cingulate | |
| | | -10,2,44 | 4.190 | 0.001 | | | Median cingulate | |
| | | -2,24,34 | 6.606 | 0.001 | | | Median cingulate | |
| | | 0,-2,34 | 4.693 | 0.001 | | | Median cingulate | |
| | | -10,10,34 | 5.615 | 0.001 | | | Median cingulate | |
| | right | 8,-30,50 | 7.182 | 0.001 | 5.85 % | -0.02, p= 0.99 | Median cingulate | 1192 |
| | | 4,-20,46 | 5.730 | 0.001 | | | Median cingulate | |
| | | 8,-16,38 | 6.052 | 0.001 | | | Median cingulate | |
| | | 4,24,36 | 6.131 | 0.001 | | | Median cingulate | |
| Dorsolateral PFC | left | -40, 42, 22 | 7.358 | 0.001 | 0.83 % | 1.32, p= 0.54 | Middle frontal gyrus | 916 |
| | right | 44, 48, 14 | 6.469 | 0.001 | 9.49 % | 1.62, p= 0.50 | Middle frontal gyrus | 1827 |
| | | 40, 42, 28 | 6.356 | 0.001 | | | Middle frontal gyrus | |
| Ventrolateral PFC | left | -38, 8 10 | 7.409 | 0.001 | 19.72 % | 2.00, p= 0.45 | Inferior frontal gyrus, opercular part | 601 |

| | | | | | | | | |
|---|---|---|---|---|---|---|---|---|
| | | -54, 12, 14 | 5.539 | 0.001 | | | Inferior frontal gyrus, opercular part, BA 44 | |
| | | -54, 12, 2 | 5.486 | 0.001 | | | Inferior frontal gyrus, opercular part, BA 44 | |
| | | -52, 12, 10 | 5.427 | 0.001 | | | Inferior frontal gyrus, opercular part, BA 44 | |
| | | -56, 8, 22 | 6.915 | 0.001 | | | Inferior frontal gyrus, opercular part, BA 44 | |
| | | -46, 44, 4 | 4.943 | 0.002 | 42.88 % | 3.64, p= 0.11 | Inferior frontal gyrus, triangular part | 1124 |
| | | -32, 30, 0 | 3.705 | 0.001 | | | Inferior frontal gyrus, triangular part | |
| | right | 48, 10, 2 | 7.444 | 0.001 | 52.15 % | 4.21, p= 0.01 | Inferior frontal gyrus, opercular part | 912 |
| | | 42, 8, 26 | 7.080 | 0.001 | | | Inferior frontal gyrus, opercular part | |
| | | 50, 8, 16 | 7.074 | 0.001 | | | Inferior frontal gyrus, opercular part, BA 44 | |
| | | 56, 10, 26 | 6.005 | 0.001 | | | Inferior frontal gyrus, opercular part, BA 44 | |
| | | 52, 14, 4 | 7.410 | 0.001 | | | Inferior frontal gyrus, opercular part | |
| | | 48, 38, 2 | 7.705 | 0.001 | 7.74 % | 1.51, p= 0.54 | Inferior frontal gyrus, triangular part | 1138 |
| | | 52, 22,30 | 5.955 | 0.001 | | | Inferior frontal gyrus, triangular part | |
| | | 50, 38, 16 | 6.827 | 0.001 | | | Inferior frontal gyrus, triangular part | |
| | | 52, 26, 22 | 4.554 | 0.001 | | | Inferior frontal gyrus, triangular part | |
| Orbitofrontal cortex | left | -44, 16, -6 | 6.626 | 0.001 | 42.73 % | 4.04, p= 0.04 | Inferior frontal gyrus, orbital part | 253 |
| | | -38, 18, -14 | 5.277 | 0.001 | | | Inferior frontal gyrus, orbital part | |
| | | -42, 18, -12 | 5.159 | 0.001 | | | Inferior frontal gyrus, orbital part | |
| Pre-/ postcentral | left | -60, 4, 14 | 6.436 | 0.001 | 28.92 % | 3.02, p= 0.16 | Postcentral gyrus | 1009 |
| | | -26, -40, 72 | 4.381 | 0.002 | | | Postcentral gyrus, BA 1 | |
| | | -24,-32,62 | 5.829 | 0.001 | | | Postcentral gyrus, BA 4 | |
| | | -40, -8, 48 | 8.230 | 0.001 | 2.35 % | 0.99, p= 0.66 | Precentral gyrus | 1374 |
| | | -48, 6, 20 | 7.705 | 0.001 | | | Precentral gyrus | |
| | | -50, 8, 38 | 6.166 | 0.001 | | | Precentral gyrus | |
| | | -44, -8, 52 | 7.592 | 0.001 | | | Precentral gyrus | |
| | | -44, 4, 36 | 6.832 | 0.001 | | | Precentral gyrus | |
| Operculum | left | -42, -30, 14 | 8.448 | 0.001 | 21.27 % | 3.25, p= 0.06 | Rolandic operculum | 687 |

| | | | | | | | | |
|---|---|---|---|---|---|---|---|---|
| | | -54, -10, 12 | 7.529 | 0.001 | | | Rolandic operculum | |
| | | -54, 0, 8 | 6.525 | 0.001 | | | Rolandic operculum | |
| | | -48, 4, 16 | 7.736 | 0.001 | | | Rolandic operculum | |
| SMA | left | 2, 20, 44 | 7.438 | 0.001 | 0.02 % | 0.47, p= 0.83 | Supplementary motor area | 1235 |
| | | -4, -4, 62 | 7.103 | 0.001 | | | Supplementary motor area | |
| | | -10, 0, 70 | 5.127 | 0.001 | | | Supplementary motor area | |
| Pre-/postcentral | right | 24, -38, 60 | 4.384 | 0.001 | 35.19 % | 1.78, p= 0.55 | Postcentral gyrus, BA 3 | 733 |
| | | 26, -38, 72 | 3.006 | 0.001 | | | Postcentral gyrus, BA 3 | |
| | | 24, -32, 64 | 3.552 | 0.001 | | | Postcentral gyrus, BA 4 | |
| | | 50, 6, 42 | 7.590 | 0.001 | 22.27 % | 2.98, p= 0.16 | Precentral gyrus | 1014 |
| | | 46, 0, 48 | 7.477 | 0.001 | | | Precentral gyrus | |
| | | 46, -8, 52 | 6.353 | 0.001 | | | Precentral gyrus | |
| Operculum | right | 40, -26, 18 | 7.278 | 0.001 | 30.17 % | 2.95, p= 0.19 | Rolandic operculum, area OP1 | 936 |
| | | 64, -18, 16 | 6.207 | 0.001 | | | Rolandic operculum, area OP1 | |
| | | 52, -4, 10 | 7.023 | 0.001 | | | Rolandic operculum, area OP4 | |
| | | 46, -10, 10 | 6.622 | 0.001 | | | Rolandic operculum, area OP4 | |
| SMA | right | 4, -6, 62 | 7.464 | 0.001 | 8.0 % | -0.14, p=0.97 | Supplementary motor area | 1287 |
| | | 4, 2, 64 | 7.354 | 0.001 | | | Supplementary motor area, BA 6 | |
| | | 8, -8, 64 | 7.096 | 0.001 | | | Supplementary motor area, BA 6 | |
| | | 12, -30, 50 | 7.116 | 0.001 | 0.71 % | -1.08, p= 0.70 | Paracentral lobule | 126 |
| Basal ganglia | left | -26, 12, 0 | 5.874 | 0.001 | 11.67 % | 2.46, p= 0.27 | Left putamen | 264 |
| | right | 32, -2, 10 | 8.070 | 0.001 | 2.24 % | 2.89, p= 0.10 | Right putamen | 350 |
| | | 20, 10, 0 | 4.133 | 0.001 | | | Right putamen | |
| | | 16, 4, 0 | 4.522 | 0.001 | | | Right pallidum | |
| Amygdala/ | right | 26, 0, -12 | 6.702 | 0.001 | 1.62 % | 2.24, p= 0.25 | Right amygdala | 115 |
| parahippocampus | | 24, 4, -26 | 3.351 | 0.003 | 51.27 % | 2.54, p= 0.37 | Right parahippocampal gyrus | 76 |
| Thalamus | left | -4, -8, 8 | 7.186 | 0.001 | 1.55 % | 2.26, p= 0.22 | Left thalamus temporal | 408 |
| | | -4, -24, 0 | 6.279 | 0.001 | | | Left thalamus prefrontal | |
| | right | 8, -18, 4 | 8.489 | 0.001 | 0.26 % | 2.84, p= 0.06 | Right thalamus prefrontal | 369 |
| Cerebellum | | 4, -76, -18 | 6.651 | 0.001 | 1.47 % | 0.16, p= 0.95 | Cerebellum, vermic lobule VI | 987 |
| | | 0, -50, -10 | 3.114 | 0.004 | | | Cerebellum, vermic lobule IV / V | |
| | | -2, -70, -16 | 6.232 | 0.001 | | | Cerebellum, vermic lobule VI | |
| | left | -8,-80,-20 | 5.903 | 0.001 | 5.74 % | -0.85, p= 0.76 | Cerebellum, crus I | 453 |

| | | | | | | | | |
|---|---|---|---|---|---|---|---|---|
| | | -40,-56,-34 | 2.529 | 0.009 | | | Cerebellum, crus I | |
| | | -38,-74,-22 | 2.545 | 0.038 | | | Cerebellum, crus I | |
| | | -36,-54,-32 | 2.760 | 0.007 | | | Cerebellum, crus I | |
| | | -6,-44,-12 | 3.112 | 0.004 | | | Left cerebellum, hemispheric lobule IV / V | 1250 |
| | | -10,-74,-20 | 6.350 | 0.001 | 7.38 % | 1.76, p= 0.46 | Left cerebellum, hemispheric lobule VI | |
| | | -20,-70,-22 | 5.693 | 0.001 | | | Left cerebellum, hemispheric lobule VI | |
| | | -22,-66,-24 | 5.611 | 0.001 | | | Left cerebellum, hemispheric lobule VI | |
| | | -24,-60,-28 | 4.415 | 0.002 | | | Left cerebellum, hemispheric lobule VI | |
| | | -28,-60,-26 | 4.230 | 0.002 | | | Left cerebellum, hemispheric lobule VI | |
| | | -32,-50,-30 | 2.882 | 0.007 | | | Left cerebellum, hemispheric lobule VI | |
| | right | 12,-72,-28 | 4.033 | 0.003 | 1.76 % | 3.43. p= 0.19 | Right cerebellum, crus I | 234 |
| | | 44,-52,-28 | 2.851 | 0.009 | | | Right cerebellum, crus I | |
| | | 44,-60,-26 | 2.793 | 0.009 | | | Right cerebellum, crus I | |
| | | 24,-64,-28 | 4.197 | 0.003 | 54.61 % | 4.25, p= 0.07 | Right cerebellum, hemispheric lobule VI | 1052 |
| | | 20,-66,-26 | 4.117 | 0.003 | | | Right cerebellum, hemispheric lobule VI | |
| | | 28,-62,-26 | 4.099 | 0.003 | | | Right cerebellum, hemispheric lobule VI | |
| | | 32,-68,-24 | 3.950 | 0.003 | | | Right cerebellum, hemispheric lobule VI | |
| | | 40,-66,-24 | 2.662 | 0.007 | | | Right cerebellum, hemispheric lobule VI | |
| Pons* | left | -10, -24, -28 | 2.625 | 0.009 | 17.93 % | 3.53, p= 0.07 | Left pons | 101 |

* half of the studies did not scan the brainstem
