# Supplementary material for: Common neural correlates of vestibular stimulation and fear learning: an fMRI meta-analysis
Source: J Neurol. 2023 Feb 1;270(4):1843–56. doi: 10.1007/s00415-023-11568-7 (PMC10025232; doi:10.1007/s00415-023-11568-7)
Supplement: Supplementary file 3 — Supplementary file3 (PDF 69 kb) [file 415_2023_11568_MOESM3_ESM.pdf]

Supplementary Table 2

Peak coordinates of the conjunction of vestibular and fear systems [21], $p < 0.05$.

**Cluster description**		**Local peaks and breakdown**			
	Hemisphere	MNI (x, y, z)	SDM-Z	Brain region	No. of voxels
Insula	left	-40, 6, 8	6.140	Anterior insula	909
		-38, 8, 4	6.135	Anterior insula	
		-38, 8, -6	5.502	Anterior insula	
	right	38, 20, 2	7.327	Anterior insula	809
		48, 12, -10	5.630	Anterior insula	
Orbitofrontal cortex	left	-44, 16, -6	6.626	Left inferior frontal gyrus, orbital part	191
		-42, 18, -12	5.159	Left inferior frontal gyrus, orbital part	
		-38, 18, -14	5.060	Left inferior frontal gyrus, orbital part	
Ventrolateral PFC	left	-50, 10, 2	5.651	Left inferior frontal gyrus, opercular part	488
		-32, 30, 0	3.705	Left inferior frontal gyrus, triangular part	
	right	52, 16, 4	6.250	Right inferior frontal gyrus, opercular part	474
Thalamus	left	-6, -14, 4	5.996	Thalamus, prefrontal	227
		-4, -26, 0	5.986	Thalamus, prefrontal	
		-4, -16, 8	5.831	Thalamus, prefrontal	
		-4, -22, 4	5.665	Thalamus, prefrontal	
	right	6, -16, 10	5.645	Thalamus, temporal	202
		4, -12, 6	5.376	Thalamus, temporal	
Temporal pole	right	54, 10, -8	5.706	Temporal pole, superior temporal gyrus	174
		42, 20, -20	3.301	Temporal pole, superior temporal gyrus	
Rolandic operculum	left	-46, 2, 10	5.525	Rolandic operculum	169
		-56, 10, 4	5.333	Rolandic operculum	
		-48, -2, 4	5.106	Rolandic operculum	
		-60, 4, 8	4.592	Rolandic operculum	
Basal ganglia	left	-12, 4, 0	5.393	Pallidum	189
		-24, 6 -8	5.087	Putamen	
	right	16, 4, 0	4.522	Pallidum	165
M1, PMC, SMA	left	-40, 0, 46	5.053	Precentral gyrus	488
		-52, 2, 44	4.630	Precentral gyrus	
		-42, 2, 56	4.334	Precentral gyrus	
		-32, -10, 52	4.025	Precentral gyrus	
		-44, -2, 56	3.979	Precentral gyrus	
		-4, 18, 42	6.086	Superior frontal gyrus, medial	189
		-2, 2 58	5.827	Supplementary motor area	976

		-2, 12, 44	5.730	Supplementary motor area	
		-8, 0, 68	4.670	Supplementary motor area	
	right	44, 4, 48	5.252	Precentral gyrus	563
		52, 4, 44	4.707	Precentral gyrus	
		6, 20, 42	6.485	Superior frontal gyrus, medial	98
		2, 6, 58	6.214	Supplementary motor area	887
		10, 12, 46	6.037	Supplementary motor area	
		2, -6, 48	4.690	Supplementary motor area	
		12, -4, 74	3.751	Supplementary motor area	
Cingulate cortex	left	-10, 10, 42	5.601	Median cingulate	886
		-10, 2, 44	4.190	Median cingulate	
		0, 2, 34	4.165	Median cingulate	
		0, -2, 36	4.135	Median cingulate	
		0, -14, 40	4.088	Median cingulate	
		-2, -18, 32	4.002	Median cingulate	
		0, 20, 24	3.928	Anterior cingulate	
		0, 12, 26	3.501	Anterior cingulate	
	right	4, 24, 32	5.695	Median cingulate	769
		2, -6, 42	4.661	Median cingulate	
Superior temporal/	left	-56, -42, 22	6.287	Superior temporal gyrus, area	1577
supramarginal gyrus		-52, -34, 28	6.187	Supramarginal gyrus	
		-52, -38, 28	6.128	Supramarginal gyrus	
		-60, -34, 24	5.792	Supramarginal gyrus	
		-60, -24, 14	5.135	Supramarginal gyrus, area OP1	
		-54, -26, 26	5.068	Supramarginal gyrus, area PFop	
		-56, -26, 20	4.936	Supramarginal gyrus, area PFop	
		-60, -42, 38	4.881	Inferior parietal lobule, area PF	
		-56, -52, 10	4.046	Middle temporal gyrus	
		-56, -62, 10	3.798	Middle temporal gyrus	
	right	54, -36, 18	6.326	Superior temporal gyrus	1489
		56, -36, 30	5.905	Supramarginal gyrus	
		56, -36, 26	5.815	Supramarginal gyrus, area PFcm	
		50, -40, 26	5.349	Supramarginal gyrus	
		56, -50, 4	3.858	Middle temporal gyrus	
		64, -40, 8	3.294	Middle temporal gyrus	
DLPFC	left	-36, 44, 22	5.635	Middle frontal gyrus	564
		-36, 40, 24	5.521	Middle frontal gyrus	
	right	34, 44, 30	3.953	Middle frontal gyrus	74
		32, 44, 20	3.541	Middle frontal gyrus	
		30, 46, 24	3.505	Middle frontal gyrus	
Cerebellum		2, -70, -16	4.874	Cerebellum, vermic lobule VI	160
	left	-28, -60, -26	4.230	Cerebellum, hemispheric lobule VI	264

		-28, -64, -24	4.118	Cerebellum, hemispheric lobule VI	
	right	28, -62, -26	4.099	Cerebellum, hemispheric lobule VI	214
		32, -68, -24	3.950	Cerebellum, hemispheric lobule VI	
		20, -74, -22	3.585	Cerebellum, hemispheric lobule VI	
Brainstem	right	10, -30, -26	2.967	Brainstem	37
